# A Facile Pre-Lithiated Strategy towards High-Performance Li_2_Se-LiTiO_2_ Composite Cathode for Li-Se Batteries

**DOI:** 10.3390/nano12050815

**Published:** 2022-02-28

**Authors:** Yang Xia, Zheng Fang, Chengwei Lu, Zhen Xiao, Xinping He, Yongping Gan, Hui Huang, Guoguang Wang, Wenkui Zhang

**Affiliations:** 1 College of Materials Science and Engineering, Zhejiang University of Technology, Hangzhou 310014, China; nanoshine@zjut.edu.cn (Y.X.); 2111925054@zjut.edu.cn (Z.F.); xinpinghe@zjut.edu.cn (X.H.); ganyp@zjut.edu.cn (Y.G.); hhui@zjut.edu.cn (H.H.); 2 Institute of Optoelectronic Materials and Devices, China Jiliang University, Hangzhou 310018, China; xiaozhen@cjlu.edu.cn; 3 Hengdian Group DMEGC Magnetics Co., Ltd., Dongyang 322118, China; wgg@dmegc.com.cn

**Keywords:** Li-Se batteries, Li_2_Se, LiTiO_2_, pre-lithiation, polyselenides

## Abstract

Conventional lithium-ion batteries with a limited energy density are unable to assume the responsibility of energy-structure innovation. Lithium-selenium (Li-Se) batteries are considered to be the next generation energy storage devices since Se cathodes have high volumetric energy density. However, the shuttle effect and volume expansion of Se cathodes severely restrict the commercialization of Li-Se batteries. Herein, a facile solid-phase synthesis method is successfully developed to fabricate novel pre-lithiated Li_2_Se-LiTiO_2_ composite cathode materials. Impressively, the rationally designed Li_2_Se-LiTiO_2_ composites demonstrate significantly enhanced electrochemical performance. On the one hand, the overpotential of Li_2_Se-LiTiO_2_ cathode extremely decreases from 2.93 V to 2.15 V. On the other hand, the specific discharge capacity of Li_2_Se-LiTiO_2_ cathode is two times higher than that of Li_2_Se. Such enhancement is mainly accounted to the emergence of oxygen vacancies during the conversion of Ti^4+^ into Ti^3+^, as well as the strong chemisorption of LiTiO_2_ particles for polyselenides. This facile pre-lithiated strategy underscores the potential importance of embedding Li into Se for boosting electrochemical performance of Se cathode, which is highly expected for high-performance Li-Se batteries to cover a wide range of practical applications.

## 1. Introduction

In recent years, the conventional lithium-ion batteries cannot meet the current development demand due to the limited energy density [1,2,3,4,5,6,7,8,9]. In this respect, sulfur cathode offers a high theoretical specific capacity of 1675 mA h g^−1^ when paired with lithium metal anode [10,11,12]. Ironically, lithium-sulfur batteries are mainly limited by non-conductive feature of sulfur cathode [13,14,15]. Selenium (Se), as an agnate element of sulfur, has similar charge-discharge reaction and volumetric capacity to sulfur, and the electronic conductivity of Se (1 × 10^−3^ S m^−1^) is much higher than sulfur (5 × 10^−28^ S m^−1^) [16,17]. Hence, Li-Se batteries are considered as one of the new generations of promising electrochemical energy storage devices [18,19,20,21]. However, the development of Li-Se batteries still faces many problems, such as notorious shuttle effect and volume expansion in Li^+^ insert/extract processes, which cause the loss of active material and low Coulombic efficiency. Meanwhile, the intermediate products (polyselenides) dissolve in ether-based electrolyte and further migrate to the anode side, which will not only passive the surface of lithium metal anode, but also severely reduce the cyclic performance [22,23,24,25,26,27].

Up to now, many strategies are adopted to circumvent the abovementioned concerns to facilitate the electrochemical performance of Li-Se batteries. The introduction of TiO_2_ as secondary phase for Li-S/Se batteries is demonstrated to be an effective strategy on account of its strong chemisorption of lithium polysulfides/lithium polyselenides [14,28,29,30,31]. However, the synthesis of TiO_2_/Se composite is cumbersome, and the volume expansion is still unresolvable. It is worth mentioning that the pre-lithiation of Se is a valid strategy to mitigate the volume expansion [32,33,34]. Unfortunately, the synthesis of fully lithiated Li_2_Se often involves complex processes and expensive chemical reagents, which remains huge challenges in great urgency [33,34,35]. Therefore, the key to break this predicament is the concise and efficient synthesis of fully pre-lithiated Se-based composites.

**Figure 1:**
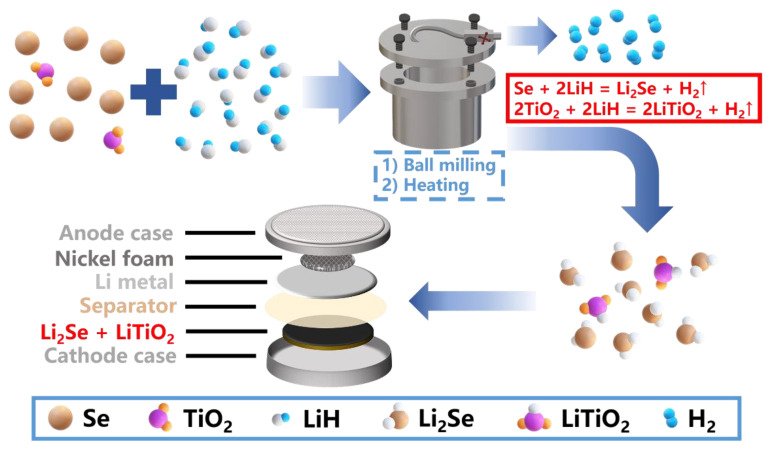
Schematic illustration of the preparation process of Li_2_Se-LiTiO_2_ composites.

In this work, as illustrated in Figure 1, we propose a novel Li_2_Se-LiTiO_2_ composite cathode, which is obtained via a two-step solid-phase method. During the synthesis process, LiH synchronously reacts with Se and TiO_2_ to in-situ form Li_2_Se-LiTiO_2_ composite and by-product H_2_ gas, which needs no subsequent separation. As the fully pre-lithiated cathode, Li_2_Se-LiTiO_2_ composite has many unique features. On the one hand, Li_2_Se could easily ameliorate the volume expansion, thereby leading to the superior structure stability and enhanced cycling stability. On the other hand, the introduced LiTiO_2_ not only could accelerate the reaction kinetics and electrochemical activities of Li_2_Se, reducing the reaction overpotential, but also offer the abundant chemical adsorption sites for tapping polyselenides, suppressing the notorious shuttle effect. Therefore, this rationally designed Li_2_Se-LiTiO_2_ composite is a promising Se-based cathode for high-performance Li-Se batteries.

## 2. Experimental Section

### 2.1. Preparation of Li_2_Se-LiTiO_2_ Composites

Selenium powder (Se, Aladdin Holdings Group Co. Ltd., Shanghai, China, purity 99.99%), anatase phase titanium dioxide (TiO_2_, Aladdin Holdings Group Co. Ltd., Shanghai, China, purity 99.8%) and lithium hydride (LiH, Aladdin Holdings Group Co. Ltd., Shanghai, China, purity 97%) were used without further purification. Li_2_Se-LiTiO_2_ samples were synthesized via two-step solid-phase reaction. In detail, Se, TiO_2_ and LiH powders were transferred into stainless-steel milling jars with a molar ratio of 4:1:9. The milling jars were milled on a planetary ball mill (QM-1SP2, Nanjing university instrument factory, Nanjing, China) at 500 rpm for 20 h. After that, the precursor was heated in a custom tube reactor at 500 °C for 3 h under vacuum to obtain Li_2_Se-LiTiO_2_ composites.

### 2.2. Materials Characterization

X-ray diffraction (XRD) patterns were carried out by an X-ray powder diffractometer (X’Pert Pro, Cu Kα radiation, Rigaku Corporation, Tokyo, Japan). All the samples were sealed with Kapton film to avoid air exposure. The morphology and microstructure were observed by scanning electron microscopy (SEM, Nova Nano 450, FEI, Hillsboro, OR, USA) with an energy dispersive spectroscopy (EDS, Oxford X-Max 80, Oxford Instruments, Oxford, UK) detector and transmission electron microscopy (TEM, Tecnai G2 F30, FEI, Hillsboro, OR, USA). X-ray photoelectron spectroscopy (XPS, ESCALAB 250X, Themo, Shanghai, China) measurements were performed on an Axis Ultra DLD system (Kratos) with a monochromatic Al Kα (1486.6 eV) X-ray source. Ultraviolet-visible (UV-vis) adsorption spectra were recorded on SHIMADSU UV-2550 spectrophotometer (SHIMADSU, Chengdu, China) to assess the polyselenide adsorption ability.

### 2.3. Electrochemical Measurements

Electrochemical properties of Li_2_Se-LiTiO_2_ electrodes were conducted on CR2025-type coin cells by using lithium metal as anode. The uniform slurry was composed of 60 wt.% Li_2_Se-LiTiO_2_ composite as active material, 30 wt.% conductive carbon (Super P, SP) and 10 wt.% ethyl cellulose, which were mixed by toluene as dispersant. The electrolyte was 1.0 mol L^−1^ lithium bis(trifluoromethanesulfonyl)imide (LiTFSI) in a co-solvent of 1,3-dioxolane (DOL)/1,2-dimethoxyethane (DME) (1:1, *v*/*v*) with 2 wt.% lithium nitrate (LiNO_3_). The electrolyte dosage in each cell was 20 μL mg^−1^ of electrolyte-to-active material ratio. All the cells were assembled in an Ar-filled glove box (H_2_O < 0.1 ppm, O_2_ < 0.1 ppm). The galvanostatic charge-discharge tests were performed on a battery testing system (Shenzhen Neware Technology Co. Ltd., Shenzhen, China). The cells were firstly charged to 3.8 V at 50 mA g^−1^, and subsequently the voltage window was adjusted to 1.7–2.6 V. For cyclic voltammogram (CV) analysis, the first forward scan started from open circuit voltage to 3.8 V, and a backward scan ended to 1.7 V. The successive scans were in the voltage range of 1.7–2.6 V at a scan rate of 0.1 mV s^−1^ on CHI650B electrochemical workstation (Chenhua, Shanghai, China). Electrochemical impedance spectra (EIS) were recorded in the frequency range from 0.1 Hz to 1.0 MHz on CHI650B electrochemical workstation.

## 3. Results and Discussion

**Figure 2:**
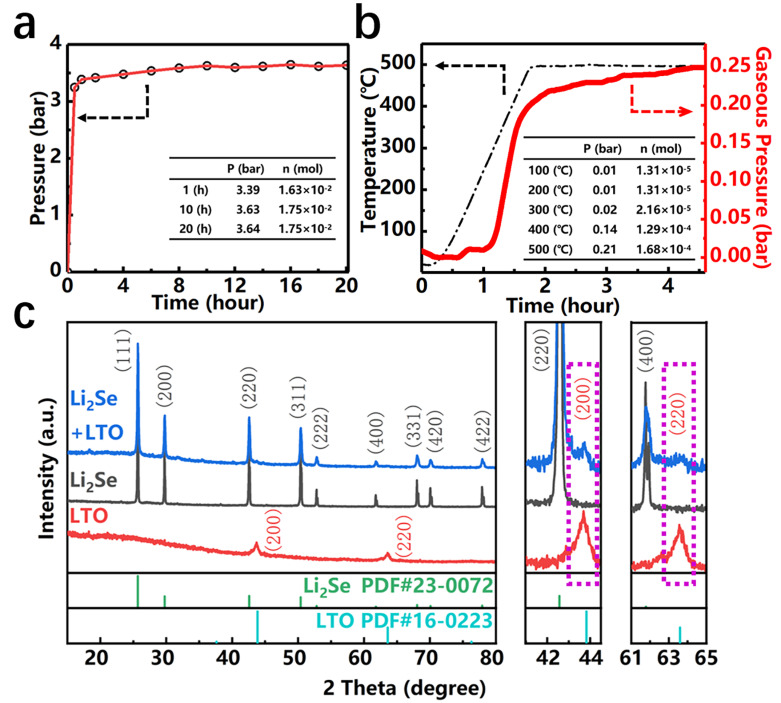
(**a**) Time-pressure curve during ball milling process. The insert table is the pressure and the number of moles in the gas at certain sampling points. (**b**) Time-temperature and time-pressure curves during heating process. The insert table is the pressure and the number of moles in the gas at certain sampling points. (**c**) XRD patterns of Li_2_Se-LiTiO_2_, Li_2_Se and LiTiO_2_.

In order to reveal the reaction mechanism of Li_2_Se-LiTiO_2_ composite by reacting LiH with Se and TiO_2_, Figure 2a,b illustrate the time-pressure curves for the ball-milling and heating processes, respectively. During the initial ball-milling process, the pressure increases dramatically to 3.39 bar within 1 h, suggesting the reaction generates a large amount of gas. Subsequently, the pressure slowly raises to 3.63 bar from 1 h to 10 h. After 10 h, the gas is no longer produced, indicating the ball-milling reaction is completed. Meanwhile, during the heating process, a conspicuous variation in pressure can be detected, in which the pressure is rapidly increased in the temperature ranging from 250 °C to 400 °C. This result implies that LiH reacts with Se and TiO_2_ in this temperature range. In order to prove the phase transformation after ball-milling and heating processes, the corresponding XRD patterns of Li_2_Se-LiTiO_2_, Li_2_Se and LiTiO_2_ are depicted in Figure 2c. The diffraction peaks of Li_2_Se-LiTiO_2_ composite shows a superposition of Li_2_Se (PDF#23-0072) and LiTiO_2_ (PDF#16-0223). It is worth mentioning that the relative intensity of the diffraction peaks of LiTiO_2_ in Li_2_Se-LiTiO_2_ composite becomes stronger with increasing proportion of LiTiO_2_ (Figure S1). According to our previous work [34], LiH could react with Se to generate Li_2_Se (2LiH + Se = Li_2_Se + H_2_ ↑). Analogously, LiTiO_2_ could be obtained by reacting LiH with TiO_2_ (2LiH + 2TiO_2_ = 2LiTiO_2_ + H_2_ ↑), which is verified by XRD results in Figure 2c and Figure S1. The distinct characteristic peaks of LiTiO_2_ match well with the cubic phase (Fm3m space group) with lattice parameters of a = b = c = 4.140 Å, which Li and Ti ions are octahedrally coordinated by O [36,37,38]. The comparison of our work with other recent studies on Li-Se batteries is shown in Table S1. On the on hand, the introduction of TiO_2_ alone could improve the cycling performance, but could not relieve the volume expansion. On the other hand, the traditional pre-lithiation process is excessively complex, and a large number of toxic solvents are used in the preparation process, which seriously pollutes the environment. The synthetic route we adopted is simple, green and environmentally friendly, which has great application prospects.

**Figure 3:**
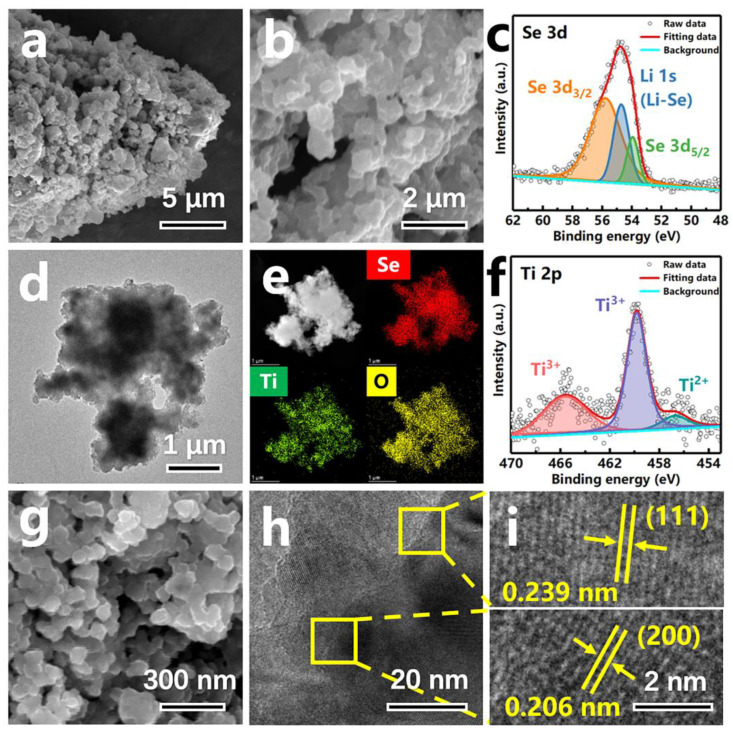
(**a**,**b**) SEM images of Li_2_Se-LiTiO_2_. (**c**,**f**) High-resolution XPS spectra of Se 3d and Ti 2p of Li_2_Se-LiTiO_2_. (**d**) TEM image of Li_2_Se-LiTiO_2_. (**e**) EDS mapping of Li_2_Se-LiTiO_2_. (**g**) SEM image of LiTiO_2_. (**h**) TEM image of LiTiO_2_. (**i**) HRTEM images of LiTiO_2_.

The microstructure, morphology and elemental distribution of Li_2_Se-LiTiO_2_ are further investigated by SEM, TEM and EDS. Compared to pristine Li_2_Se (Figure S2a), Li_2_Se-LiTiO_2_ composite has more regular morphology with small particle size ranging from 60 to 100 nm (Figure 3a,b), which will be favorable to significantly enhance the electrochemical reactivity and structural stability [34]. Additionally, as illustrated in Figure 3d,e, Se signal is uniformly distributed in Li_2_Se-LiTiO_2_ composite, whereas Ti and O signals are tightly conjunct with each other. This result vividly demonstrates Li_2_Se has a good distribution in LiTiO_2_. Moreover, the surface chemical state of Li_2_Se-LiTiO_2_ composite is elucidated by XPS test. As shown in Figure 3c,f, the peaks of Se-3d and Ti-2p both slightly shift towards the high binding energy since some chemotactic bonds are formed between Li_2_Se and LiTiO_2_, which will weaken the shielding effect of the electron atmosphere. It is worth noting that compared to commercial anatase TiO_2_ (Figure S2e), LiTiO_2_ (Figure 3g) has slightly larger particle size in the range of 60–80 nm with rougher surface and particle agglomeration. This is attributed to the strong collision of TiO_2_ particles during the synthesis process. Additionally, the lattice spacings of TiO_2_ (Figure S2f,g) are 0.148 nm and 0.149 nm, respectively, corresponding to the (204) and (213) interplanar distances of anatase TiO_2_ phase (PDF#21-1272). In contrast, the lattice distances are 0.239 nm and 0.206 nm as shown in Figure 3i, respectively, indexing the (111) and (200) crystal planes of LiTiO_2_ (PDF#16-0223), thereby further providing the evidence of the existence of LiTiO_2_ in Li_2_Se-LiTiO_2_ composite.

**Figure 4:**
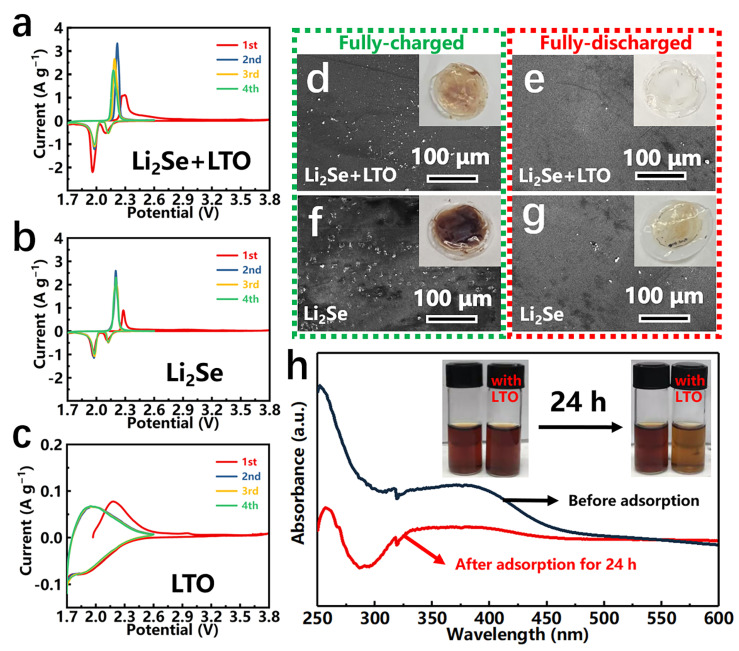
(**a**–**c**) CV profiles of Li_2_Se-LiTiO_2_, Li_2_Se and LiTiO_2_ cathodes. (**d**–**g**) SEM images of Li_2_Se-LiTiO_2_ and Li_2_Se cathodes at fully charged/discharged state. The insets are the digital photos of separators. (**h**) UV-vis spectra of Li_2_Se_6_ solution with LiTiO_2_ before/after adsorption test.

Figure 4a–c presents the CV profiles of Li_2_Se-LiTiO_2_, Li_2_Se and LiTiO_2_ cathodes in the voltage range of 1.7–3.8 V at a scan rate of 0.1 mV s^−1^. At the first Li^+^ extraction step, Li_2_Se-LiTiO_2_ cathode has two adjacent oxidation peaks at 2.27 V and 2.31 V. According to previous works [17,21,39], the oxidation peaks of LiTiO_2_ and Li_2_Se are generally located at 2.18 V (Figure 4c) and 2.31 V (Figure 4b), which are assigned to the conversion of Ti^3+^ to Ti^4+^ in LiTiO_2_ and the formation of hexagonal element Se^0^ in Li_2_Se, respectively. Meanwhile, two-stage processes are observed during the first lithiation step, corresponding to the reduction from Se to polyselenides (2.05 V) and then to Li_2_Se/Li_2_Se_2_ (1.85 V) [19,34]. Noteworthy, the position of reduction peaks after the first cycle slightly moves to the higher potential, implying the overpotential is decreased. This is mainly due to the lower de-lithiated potential of LiTiO_2_, which triggers the Li^+^ extraction of Li_2_Se.

To further verify the role of LiTiO_2_ in the Li^+^ insert/extract processes of Li_2_Se, the color change of separators is examined by digital photos and SEM images. As shown in Figure 4d, in the fully charged state, the separator of Li_2_Se-LiTiO_2_ turns light reddish-brown, as well as only a few of particles are observed at the surface of separator. In contrast, the separator of Li_2_Se turns conspicuous brick-red, and many particles appear on the surface of separator as illustrated in Figure 4f. Meanwhile, during the fully discharged state, it shows the similar phenomenon, which the separator of Li_2_Se-LiTiO_2_ sample is much cleaner than that of Li_2_Se sample (Figure 4e,g). Apparently, LiTiO_2_ facilitates the conversion of Li_2_Se to Se, and it inhibits the dissolution of polyselenides.

Generally, TiO_2_ has good chemisorption on polysulfides/polyselenides for Li-S and Li-Se batteries [14,28,29,30,31]. In this work, LiTiO_2_ is a fully lithiated state of TiO_2_, which has very different chemical state to TiO_2_. Therefore, it is necessary to inspect the polyselenide adsorption capability of LiTiO_2_. As depicted in Figure 4h, we design the simulated polyselenide adsorption experiment, which LiTiO_2_ is added to the simulative Li_2_Se_6_ solution. After 24 h, the color of Li_2_Se_6_ solution changes from dark brown to light brown with adding LiTiO_2_. Moreover, on the basis of UV-vis result, the characteristic peak intensity is dramatically decreased, reflecting the reduced concentration of Li_2_Se_6_ in simulated solution. This result clearly indicates that LiTiO_2_ has superior polyselenides trapping ability, which is favorable to achieve stable electrochemical performance of Li-Se batteries.

**Figure 5:**
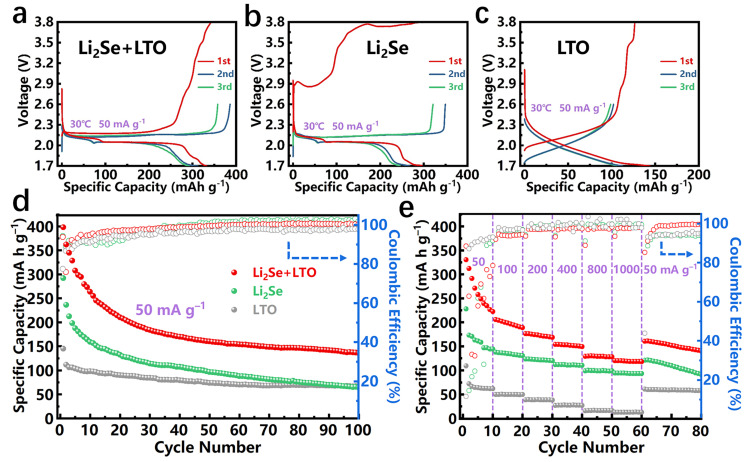
(**a**–**c**) The initial charge-discharge profiles of Li_2_Se-LiTiO_2_, Li_2_Se and LiTiO_2_ electrodes at a current density of 50 mA g^−1^. (**d**,**e**) Cycling stability at a current density of 50 mA g^−1^ and multi-rate cycling performance of Li_2_Se-LiTiO_2_, Li_2_Se and LiTiO_2_ electrodes.

The electrochemical performance of Li_2_Se-LiTiO_2_ composite is evaluated by coin cells. Figure 5a–c exhibit the galvanostatic charge-discharge profiles of Li_2_Se-LiTiO_2_, Li_2_Se and LiTiO_2_ cathodes. A noticeable overpotential (2.93 V) can be observed in Li_2_Se cathode during the first charge profile, which is related to the obstruction of Li^+^ extraction from crystalline Li_2_Se and the formation of a new interface [40]. Impressively, the overpotential of Li_2_Se-LiTiO_2_ cathode is dramatically diminished to only 2.15 V. It is probably attributed to the presence of oxygen vacancies, where Ti^4+^ is converted to Ti^3+^ in the synthesis process [41,42,43]. In the charging process, these oxygen vacancies stabilize the free ions, acting as Lewis acid sites that can interact strongly with polyselenides and release more Li^+^ [44,45]. Meanwhile, the overpotential of Li_2_Se-TiO_2_ cathode is still present (Figure S3a), which further supports our assumptions. In the subsequent charging process, a long plateau at ~2.18 V is observed, which is related to the reversible conversion of Li_2_Se_2_/Li_2_Se to Li_2_Se*_n_* (8 ≥ *n* ≥ 4) and Se [46,47]. Meanwhile, during the discharge process, the Li_2_Se, Li_2_Se-LiTiO_2_ and Li_2_Se-TiO_2_ cathodes have two voltage plateaus located at ~2.12 V and ~2.05 V, respectively, which are ascribed to the multistep phase transitions of Se to soluble long-chain Li_2_Se*_n_* (8 ≥ *n* ≥ 4) and further to insoluble short-chain Li_2_Se_2_/Li_2_Se [48,49]. It is worth noting that when the first charging voltage is 2.6 V, Li_2_Se is nonactivated and the Li^+^ is not completely detached from Li_2_Se, exhibiting terrible cycling performance (Figure S4).

For the purpose to inspect the long-term cycling stability and multi-rate ability of Li_2_Se-LiTiO_2_ cathode, Li_2_Se and LiTiO_2_ cathodes are employed as counterparts. As shown in Figure 5d, Li_2_Se-LiTiO_2_ cathode delivers a high initial discharge capacity of 398 mA h g^−1^, which are higher than that of Li_2_Se cathode (292 mA h g^−1^). Noteworthy, the capacity fading of Li_2_Se is 19% after 1 cycle, whereas Li_2_Se-LiTiO_2_ cathode is only 9%. The reversible specific capacity of Li_2_Se-LiTiO_2_ cathode still remains at 134 mA h g^−1^ after 100 cycles. In sharp contrast, the specific discharge capacity of Li_2_Se cathode rapidly decays to 65 mA h g^−1^ after 100 cycles. Additionally, Li_2_Se-LiTiO_2_ cathode exhibits the better multi-rate capability compared to Li_2_Se cathode, which delivers reversible capacities of 330, 206, 176, 154, 129 and 120 mA h g^−1^ with upward current densities of 50, 100, 200, 400, 800 and 1000 mA g^−1^, respectively. Notably, when the current density is returned to 50 mA g^−1^, a reversible capacity of 160 mA h g^−1^ is recovered. It should be mentioned that the initial discharge capacity of LiTiO_2_ is 109 mA h g^−1^, and it delivers 57 mA h g^−1^ after 100 cycles. However, the LiTiO_2_ content in composite is 20%, thereby contributing a small capacity in Li_2_Se-LiTiO_2_ cathode. The conductive carbon in the cathode provides few capacities (Figure S5). When the molar ratio of Li_2_Se to LiTiO_2_ is 7 to 3, the discharge capacity of Li_2_Se-LiTiO_2_ cathode drops rapidly to only 152 mA h g^−1^ after the 1st cycle, which is related to the high content of LiTiO_2_ with low specific capacity. Moreover, the cycling stability and multi-rate performance comparison of Li_2_Se-LiTiO_2_ composites with various LiTiO_2_ contents are illustrated in Figure S3c. Apparently, the sample with 20% LiTiO_2_ demonstrates the best cycling stability and rate capability compared to other counterparts.

Nyquist plots further are performed to confirm the reaction kinetics and electrochemical activities of Li_2_Se-LiTiO_2_ cathode before/after cycling. As depicted in Figure S3b, Li_2_Se-LiTiO_2_ and Li_2_Se cathodes both have a semicircle in high frequency along with a sloping straight line in low frequency. Generally, the formation of soluble polyselenides during the charging/discharging process causing an irreversible loss of active material and the retention of polyselenides in the electrolyte, thus inhibiting the transfer of charge. After 100 cycles, Li_2_Se-LiTiO_2_ cathode has the smaller semicircle, corresponding to the lower charge transfer resistance than Li_2_Se cathode. This is attributed to the introduction of LiTiO_2_ with strong chemisorption on polyselenides, which significantly reduces the internal resistance and further facilitates the mass transfer process.

**Figure 6:**
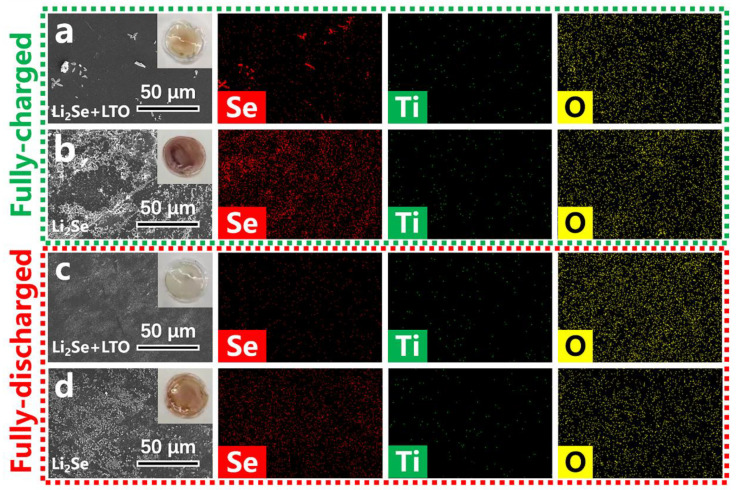
Digital photos, SEM images and EDS mapping of the separators assembled in Li_2_Se-LiTiO_2_ (**a**,**c**) and Li_2_Se (**b**,**d**) based cells at fully charged/discharged states after 10 cycles.

To further demonstrate the sustained effect of LiTiO_2_ in stabilizing cycling performance of Li_2_Se, the separators after cycling are examined by digital photos, SEM and EDS mapping (Figure 6a–d). In the fully-charged state, the diffraction peaks of Li_2_Se disappear with the conversion of Li_2_Se to Se, and the newly appeared peaks may be related to the formation of SEI layer and the production of amorphous Se (Figure S6). A large number of reddish-brown particles are observed on the separator of Li_2_Se cathode. However, only a few of particles are detected on the separator of Li_2_Se-LiTiO_2_ cathode. These particles are proved to be polyselenides by EDS mapping characterization. After fully discharging, Se combines with Li^+^ to regenerate Li_2_Se and diffraction peaks of Li_2_Se are observed again (Figure S6). Many particles still remain on the surface of the separator in Li_2_Se based cell, whereas the surface of the separator of Li_2_Se-LiTiO_2_ based cell is spotlessly clean that virtually no particles are observed. This result clearly suggests a long-term role of LiTiO_2_ to suppress the polyselenide migration and strictly confine polyselenides in the cathode side, matching well with the results in Figure 4d–g.

## 4. Conclusions

In summary, an innovative Li_2_Se-LiTiO_2_ composite cathode material is successfully developed by a two-step solid-phase method for advanced Li-Se batteries. LiTiO_2_ with strong chemical adsorption of polyselenides, emerges oxygen vacancies during the conversion of Ti^4+^ into Ti^3+^. These oxygen vacancies release of Li^+^ from polyselenide and improve the utilization of Se. Li-Se battery with this novel cathode exhibits remarkable electrochemical performance in terms of the reduced overpotential from 2.93 V to 2.15 V and high specific discharge capacity that cathode is two times higher than Li_2_Se cathode. This work provides fantastic inspiration for rationally designing fully pre-lithiated Se-based cathodes in advanced Li-Se batteries.

## Data Availability

Data can be available upon request from the authors.

## Supplementary Materials

The following supporting information can be downloaded at: https://www.mdpi.com/article/10.3390/nano12050815/s1, Table S1. The comparison of cycling performance for Li-Se batteries based on recent studies. Figure S1. XRD patterns of gradient molar ratio of LiTiO_2_ in Li_2_Se. Figure S2. (a) SEM image of Li_2_Se. (b) SEM image of LiTiO_2_. (c) High-resolution XPS spectrum of Se 3d region in Li_2_Se. (d) High-resolution XPS spectrum of Ti 2p region in LiTiO_2_. (e) SEM image of TiO_2_. (f) TEM image of TiO_2_. (g) HR-TEM images of TiO_2_. Figure S3. (a) The initial charge-discharge profiles of Li_2_Se-TiO_2_ at a current density of 50 mA g^−1^. (b) Nyquist plots before/after cycling of Li_2_Se-LiTiO_2_ and Li_2_Se. (c–d) Cycling stability at a current density of 50 mA g^−1^ and multi-rate cycling performance of gradient molar ratio of LiTiO_2_ in Li_2_Se electrodes. Figure S4. (a) The charge-discharge profiles of Li_2_Se-LiTiO_2_ at 1.7–2.6 V. (b) Cycling stability of Li_2_Se-LiTiO_2_ at 1.7–2.6 V with a current density of 50 mA g^−1^. Figure S5. The initial charge-discharge profiles of conductive carbon at a current density of 50 mA g^−1^. Figure S6. XRD patterns of Li_2_Se-LiTiO_2_ cathodes after charged/discharged.
