# Supplementary material for: A Facile Pre-Lithiated Strategy towards High-Performance Li2Se-LiTiO2 Composite Cathode for Li-Se Batteries
Source: Nanomaterials (Basel). 2022 Feb 28;12(5):815. doi: 10.3390/nano12050815 (PMC8912804; doi:10.3390/nano12050815)
Supplement: Supplementary file 1 [file nanomaterials-12-00815-s001.zip › nanomaterials-1607261-supplementary.pdf]

Supplementary Materials

A Facile Pre-Lithiated Strategy towards High-Performance Li_2Se-$LiTiO_2$ Composite Cathode for Li-Se Batteries

Yang Xia [1], Zheng Fang [1], Chengwei Lu [1,*], Zhen Xiao [2], Xinping He [1], Yongping Gan [1], Hui Huang [1], Guoguang Wang [3] and Wenkui Zhang [1,*]

[1] College of Materials Science and Engineering, Zhejiang University of Technology, Hangzhou 310014, China; nanoshine@zjut.edu.cn (Y.X.); 2111925054@zjut.edu.cn (Z.F.); xinpinghe@zjut.edu.cn (X.H.); ganyp@zjut.edu.cn (Y.G.); hhui@zjut.edu.cn (H.H.)
[2] Institute of Optoelectronic Materials and Devices, China Jiliang University, Hangzhou 310018, China; xiaozhen@cjlu.edu.cn
[3] Hengdian Group DMEGC Magnetics Co., Ltd., Dongyang 322118, China; wgg@dmegc.com.cn
* Correspondence: lcw5460@zjut.edu.cn (C.L.); msechem@zjut.edu.cn (W.Z.)

Table S1. The comparison of cycling performance for Li-Se batteries based on recent studies.

Materials	2D-$MoSe_2$	Li_2Se	C-Li_2Se@C	Li_2Se-$LiTiO_2$
Synthesis method	1) multiple re-fluxes 2) extraction 3) heating	1) ball-milling 2) heating	1) chemical reaction between Se powder and Li super hydride solution 2) evaporated EtOH and NMP 3) carbonized	1) ball-milling 2) heating
Reaction condition	200 °C	500 °C	700 °C	500 °C
Specific capacity (mA h g^{-1})	715	698	~300	397
Coulombic efficiency	97.5%	98.9%	99.2%	99.7%
Reference	[1]	[2]	[3]	This work

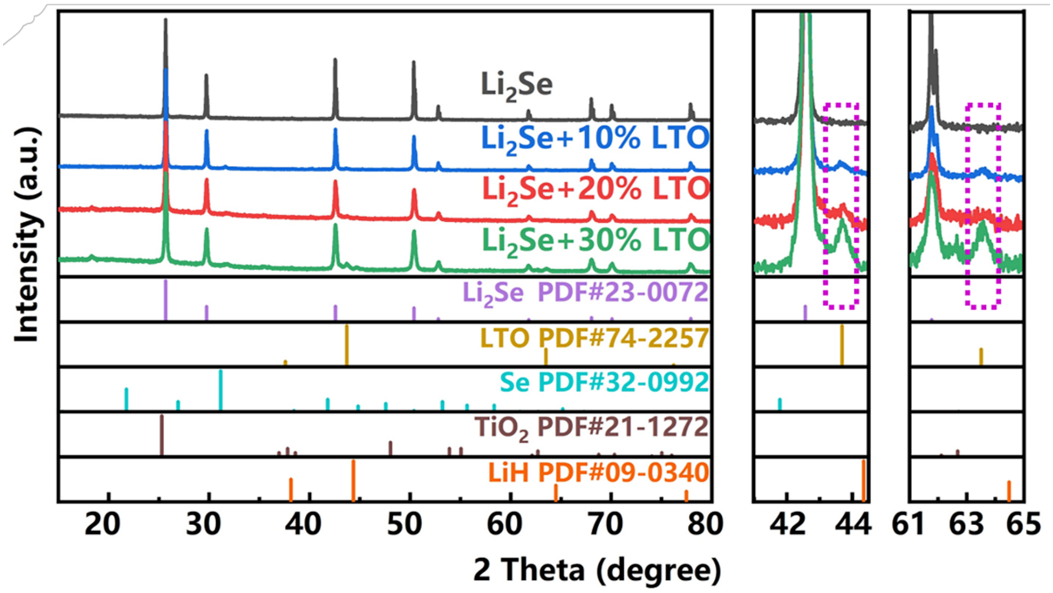

Figure S1. XRD patterns of gradient molar ratio of $LiTiO_2$ in Li_2Se.

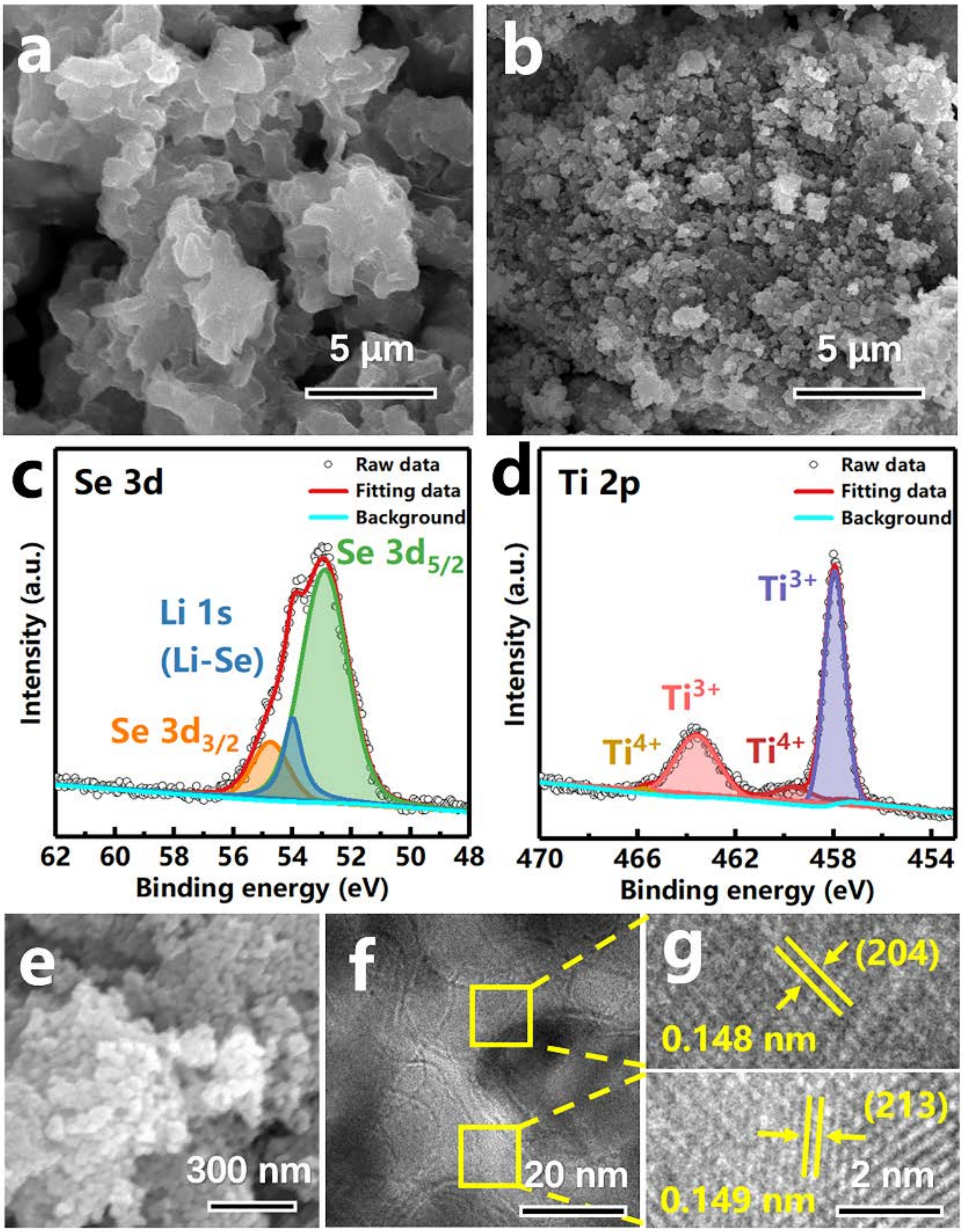

Figure S2. (**a**) SEM image of Li_2Se. (**b**) SEM image of $LiTiO_2$. (**c**) High-resolution XPS spectrum of Se 3d region in Li_2Se. (**d**) High-resolution XPS spectrum of Ti 2p region in $LiTiO_2$. (**e**) SEM image of TiO_2. (**f**) TEM image of TiO_2. (**g**) HR-TEM images of TiO_2.

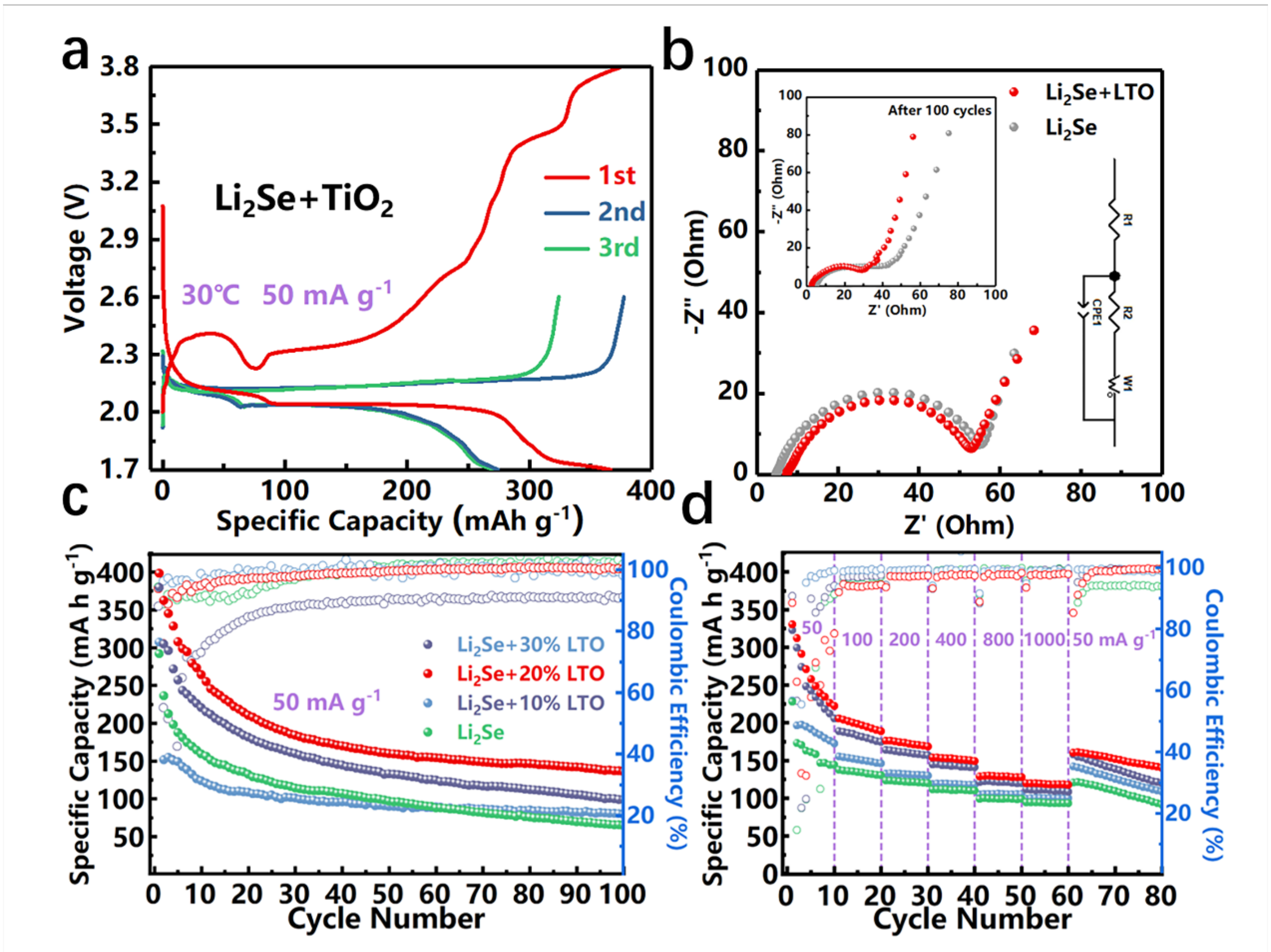

Figure S3. (**a**) The initial charge-discharge profiles of Li_2Se-TiO_2 at a current density of 50 mA g^{-1}. (**b**) Nyquist plots before/after cycling of Li_2Se-$LiTiO_2$ and Li_2Se. (**c**–**d**) Cycling stability at a current density of 50 mA g^{-1} and multi-rate cycling performance of gradient molar ratio of $LiTiO_2$ in Li_2Se electrodes.

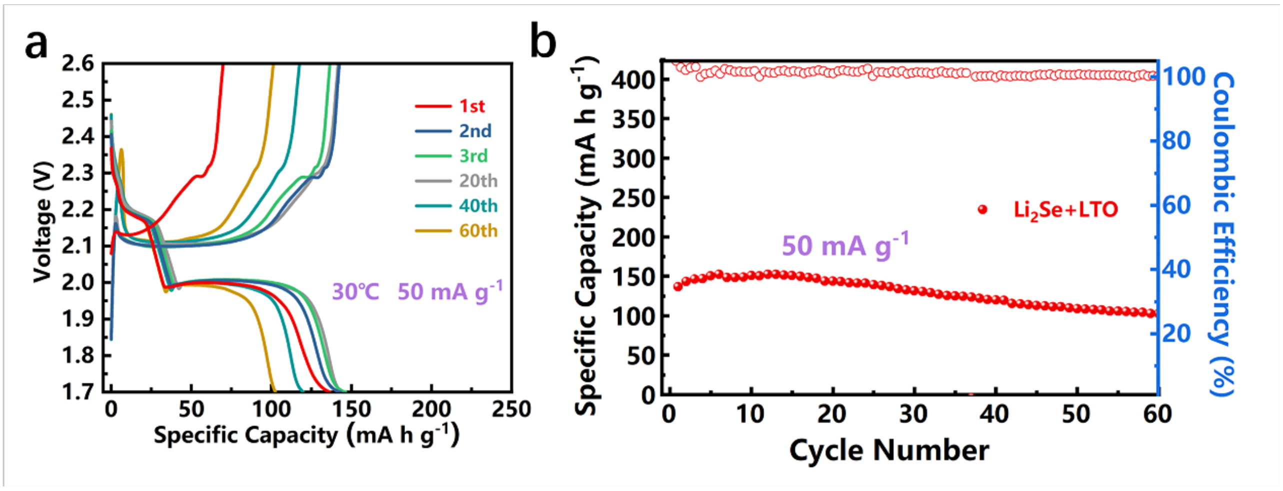

Figure S4. (a) The charge-discharge profiles of Li_2Se-$LiTiO_2$ at 1.7–2.6 V. (b) Cycling stability of Li_2Se-$LiTiO_2$ at 1.7–2.6 V with a current density of 50 mA g^{-1}.

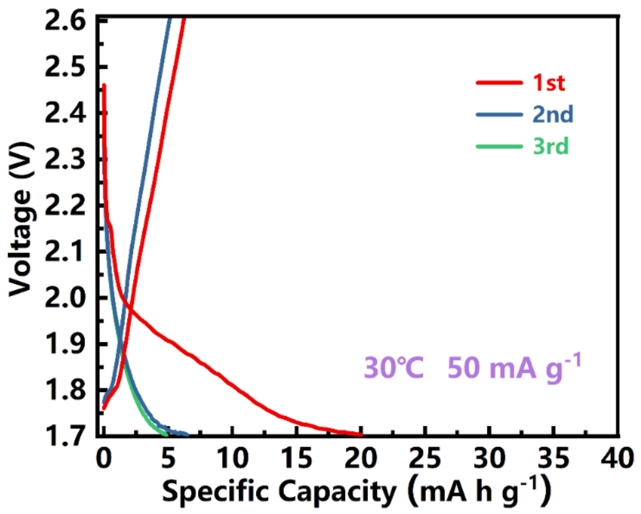

Figure S5. The initial charge-discharge profiles of conductive carbon at a current density of 50 mA g^{-1}.

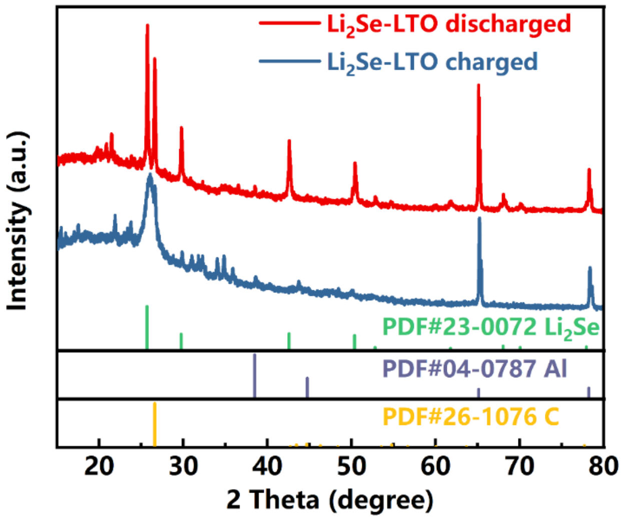

Figure S6. XRD patterns of Li_2Se-$LiTiO_2$ cathodes after charged/discharged.

References

[1] Bui, H.T.; Jang, H.; Ahn, D.; Han, J.; Sung, M.; Kutwade, V.; Patil, M.; Sharma, R.; Han, S.-H. High-performance Li–Se battery: Li_2Se cathode as intercalation product of electrochemical in situ reduction of multilayer graphene-embedded 2D-$MoSe_2$. Electrochim. Acta 2020, 368, 137556. https://doi.org/10.1016/j.electacta.2020.137556.

[2] Lu, C.; Zhang, W.; Fang, R.; Xiao, Z.; Huang, H.; Gan, Y.; Zhang, J.; He, X.; Liang, C.; Zhu, D.; et al. Facile and efficient synthesis of Li_2Se particles towards high-areal capacity Li_2Se cathode for advanced Li–Se battery. Sustain. Mater. Technol. 2021, 29, e00288. https://doi.org/10.1016/j.susmat.2021.e00288.

[3] Wu, F.; Lee, J.T.; Xiao, Y.; Yushin, G. Nanostructured Li_2Se cathodes for high performance lithium-selenium batteries. Nano Energy 2016, 27, 238–246. https://doi.org/10.1016/j.nanoen.2016.07.012.
